# Inside the Hospitalization Voyage of Schizophrenia Care: A Single-Center Journey

**DOI:** 10.3390/medicina60081214

**Published:** 2024-07-26

**Authors:** Răzvan Pop, Cătălina Angela Crișan, Ioana Valentina Micluția, Emilia Pop, Mihaela Iancu, Sorana D. Bolboacă

**Affiliations:** 1 Medical Informatics and Biostatistics, Department 11—Medical Education, Faculty of Medicine, “Iuliu Hațieganu” University of Medicine and Pharmacy, Louis Pasteur Str., No. 6, 400349 Cluj-Napoca, Romania; pop.razvan.florin@elearn.umfcluj.ro (R.P.); miancu@umfcluj.ro (M.I.); 2 Department of Neurosciences, “Iuliu Hațieganu” University of Medicine and Pharmacy, Victor Babeș Str., No. 43, 400012 Cluj-Napoca, Romania; 3 Children and Adolescent Psychiatry Clinic, Clinical Emergency Hospital for Children, Republicii Str., No. 57, 400489 Cluj-Napoca, Romania

**Keywords:** schizophrenia, repeated hospitalizations, psychometry

## Abstract

Schizophrenia poses significant challenges for individuals and caregivers, often leading to recurrent hospitalizations. Limited information on patients with schizophrenia and multiple hospitalizations in Romania is available in the scientific literature. Our study aimed to evaluate the characteristics of patients with schizophrenia with multiple hospitalizations in a single center in Cluj-Napoca, analyzing if specific patterns exist between patients with two or more hospitalizations or between men and women. We conducted a retrospective study on patients diagnosed with schizophrenia according to the 10th revision of the International Classification of Diseases (ICD 10), hospitalized at the County Emergency Hospital of Cluj-Napoca, Romania, between 2018 and 2022. Data on demographics, somatic comorbidities, symptom severity using the positive and negative syndrome scale (PANSS) or the brief psychiatric rating scale (BPRS), antipsychotic medication, and adverse effects were collected. We evaluated 62 patients, aged from 23 to 57 years, with 157 hospitalizations (ranging from two to seven per patient). No familial history of schizophrenia (56.5%) or bipolar disorder (71%) was reported by most patients. Forty-eight patients were male, and 45 had two hospitalizations. Age, sex, living place and conditions, season of birth, and marital status were similar in patients with two or more than two hospitalizations (*p*-values > 0.10). Significant differences were observed between patients with two or more than two hospitalizations regarding smoking (63.3% vs. 79.1%, *p*-value = 0.0029) and symptoms of fear at admission (40.0% vs. 65.7%, *p*-value = 0.0015). We observed lower scores in the overall PANSS and BPRS scores at discharge compared to admission (*p*-values < 0.001), regardless of the group (two or more than two hospitalizations, men vs. women). Men and women showed differences in hospitalization stays (median 17.25 vs. 15 days, *p*-value < 0.001) and BPRS scores at admission (*p*-value = 0.012) and discharge (*p*-value = 0.016). Fewer First-Generation Antipsychotics were prescribed for those with two admissions, and nearly half reported adverse effects, notably tachycardia (29%), with similar occurrence within groups. Our results showed that the candidate for multiple hospitalizations is a male, with a mean age of 37 years, unmarried, and living with someone in urban settings, more likely a smoker who exhibits fear symptoms.

## 1. Introduction

Schizophrenia is a severe, chronic, and disabling psychiatric disease that causes functioning impairment in one or more essential domains of independence such as professional, interpersonal relationships, or self-care. Globally, prevalent cases increased from 13.1 million in 1990 to 20.9 million in 2016 [1]. Epidemiological studies show a global lifetime prevalence between 7.2 and 7.49 per 1000 individuals (2020 data) [2,3]. According to the World Health Organization, schizophrenia impacts around 24 million individuals globally, equating to approximately 1 in 300 people (0.32%). Among adults, this rate rises to 1 in 222 individuals (0.45%). Schizophrenia typically emerges during late adolescence or early twenties, with the onset occurring earlier in men than in women. Individuals with schizophrenia face a significantly elevated risk of premature mortality, being 2 to 3 times more likely to die early compared to the general population. Experts frequently attribute this heightened mortality risk to physical health conditions such as cardiovascular diseases, metabolic disorders, and infectious illnesses (2022 data) [4]. The annual costs, both direct and indirect, for managing patients with schizophrenia range from 94 million dollars to 102 billion dollars, as reported in a study published in 2016 [5]. Due to its chronic progressive nature and the predominant lack of insight, patients with schizophrenia exhibit a recurrent pattern regarding the frequency of hospitalizations. Researchers have shown that both psychopharmacological and psychological interventions substantially alleviate symptoms of schizophrenia. Despite the available treatments, the condition remains closely linked with a high frequency of hospital admissions and prolonged utilization of psychiatric services [6,7]. Golay et al. highlight the recurrent pattern of hospitalizations among patients with schizophrenia compared to other psychiatric diagnoses, such as depression, anxiety and stress-related disorders, personality disorders, alcohol-related disorders, dementia, and drug-related disorders [8]. Tal and Shaul demonstrated on a population from Israel statistically significant differences between patients with schizophrenia without comorbidities, and those with one, two, or three associated comorbidities, such as substance use disorder, personality disorder, organic mental disorders, intellectual disability, and obsessive-compulsive disorder, regarding hospitalization stay and annual recurrent hospitalization [9]. Regarding the median annual number of hospitalizations for patients with one, two, or three or more additional diagnoses, the figures rose to 0.49 (95% CI: 0.44–0.54), 0.71 (95% CI: 0.65–0.84), and 1.11 (95% CI: 0.85–1.53), compared to patients with no comorbidity (0.22, 95% CI: 0.21 to 0.23). Additionally, the presence of other diseases led to an increase in the median duration of hospital stays per year (from 18.06 days (95% CI: 16.05–19.78)). The study also emphasized that as the number of additional diagnoses increased, so did the percentage of patients who visited the emergency room (from 51.9% for absence of any comorbidity, 64.2% with one, 77.3% for patients with two, and 90.2% for patients with three or more additional diagnoses) [9].

The scientific literature reflecting Romanian patients with schizophrenia is scarce. In 2018, the reported incidence of mental health problems was 14.3% in Romania, with bipolar disorders and schizophrenia weighing 1.06% [10]. The number of cases with schizophrenia in Romania was 50,068 as reported in 2016 [11], without any other statistics. Schizophrenia in Romania accounted for 0.04% of total deaths as reported in 2020 [12]. A national mental health report in 2023 estimates the total costs for managing patients with schizophrenia at approximately USD 441 million [13]. Rad et al. [14] reported on a Romanian cohort significant characteristic regarding the evolution of psychosis manifested in childhood and adolescence, namely notable associations between early-onset psychosis, with 14.74% of participants experiencing onset before 13 years old, and a higher frequency of relapses and hospitalizations in adult psychiatry. Furthermore, a statistically significant correlation was identified between familial psychiatric history and an increased frequency of relapses and hospitalizations, with 57.4% of the subjects having a family history of psychiatric disorders [14]. Regarding mental health-related stigma in Romania, Manescu et al. [15] reported slightly higher levels of stigmatizing attitudes towards individuals with mental illness in the Romanian population compared to the European average. Prevalent stereotypes included perceptions of unpredictability and dangerousness, held by 65% and 45% of the population, respectively. Additionally, a smaller proportion believed individuals with mental illness never recover (20%) and are solely responsible for their condition (14%). The diagnosis of schizophrenia in Romania adheres to the diagnostic criteria outlined in the 10th edition of the International Classification of Diseases (ICD-10). However, the Romanian psychopharmacological treatment guideline is missing, a fact highlighted by Stevović et al. [16]. Iaru et al. [17] reported the utilization patterns of anxiolytics, antidepressants (ADs), and antipsychotics (APs) in Romania during 1998–2018 and underscore a substantial increase. Specifically, the usage of antidepressants (ADs) has seen a noteworthy surge, exhibiting an average annual growth rate of 13.7% since 1999 [17]. Similarly, the utilization of antipsychotics (APs) has demonstrated a consistent upward trend from 2003 to 2018 [17]. Moreover, a noticeable rise in using long-acting formulations of APs during the latter years of analysis (2015–2018) is observed, suggesting an increasing inclination towards their adoption [17]. Regarding the attributes of patients experiencing multiple hospital admissions in Romania, our previous study [18] examining the predictors of clinical progression, which involved a cohort of 80 patients with hospitalizations up to 35, underscores that the level of insight and PANSS score collectively explained only 16% of the variability in the amelioration of psychotic symptoms during hospital stays.

Horosan et al. [19] evaluated a cohort of patients with schizophrenia and multiple hospitalizations in one clinic in Bucharest and reported a higher overall PANSS score as a risk factor for re-hospitalizations. To the best of our knowledge, no other reports are available in the scientific literature reflecting the patients with schizophrenia and multiple hospitalizations in Romania. Our study aimed to evaluate the characteristics of patients with schizophrenia with multiple hospitalizations in a single center in Cluj-Napoca, analyzing if specific patterns exist between patients with two or more hospitalizations or between men and women.

## 2. Materials and Methods

Following the 1964 Declaration of Helsinki, our research was approved by the ethics committee of “Iuliu Hațieganu” University of Medicine and Pharmacy Cluj-Napoca, Romania (approval number 21 from 27 January 2023), and by the ethics committee of the County Emergency Hospital of Cluj-Napoca, Romania (application number 7530 from 16 February 2023).

### 2.1. Study Design

**Figure 1:**
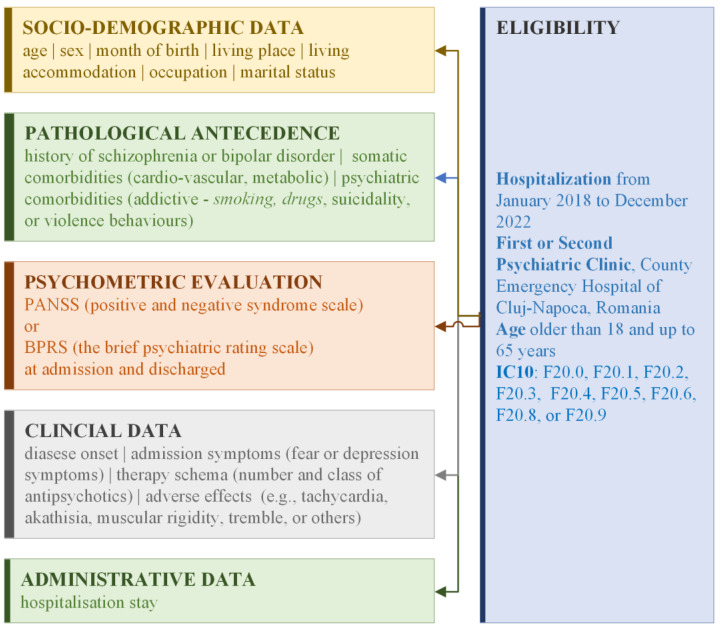
Flowchart reflecting the study cohort: eligibility and data collection.

We conducted a retrospective study on patients’ medical records hospitalized during January 2018 and December 2022 at the First or Second Psychiatric Clinics of the County Emergency Hospital of Cluj-Napoca, Romania. The eligible patients were those diagnosed according to the ICD10 with paranoid schizophrenia (F20.0), hebephrenic schizophrenia (F20.1), catatonic schizophrenia (F20.2), undifferentiated schizophrenia (F20.3), post-schizophrenic depression (F20.4), residual schizophrenia (F20.5), simple schizophrenia (F20.6), other schizophrenia (F20.8), or schizophrenia, unspecified (F20.9) (Figure 1). The inclusion criteria consisted of patients with the primary diagnostic of schizophrenia, aged between 18 and 65 years, who were evaluated at both admission and discharge using either the PANSS/SCI-PANSS (structured clinical interview for the positive and negative syndrome) or BPRS (brief psychiatry rating scale) scale. Patients with diagnoses of mental retardation, bipolar disorder, schizoaffective disorder, or persistent delusional disorder were excluded. Furthermore, patients hospitalized according to the Mental Health law of lacked treatment descriptions were also excluded.

Initially, patients were searched for in the hospital electronic database by selecting the filter for more than one hospitalization and the age range of 18 and 65 years, within the specified period. Subsequently, using the registration number of the observation sheet, we accessed their medical records in the archive of the Psychiatry Clinic at the Cluj-Napoca County Hospital. The data were recorded in accordance with the informed consent agreement regarding voluntary participation in the educational and research process signed by the patient upon admission, and their anonymized information was entered into a Microsoft Excel for Windows (Microsoft 365, Microsoft Corporation, WA, USA) database using a password-protected file.

### 2.2. Psychometric Evaluation

The psychometric evaluation of patients regarding the type of questionnaire was carried out based on the preferences of the attending physician. A summary of the questionnaires used in the psychometric evaluation is presented in Table 1.

### 2.3. Statistical Analysis

We performed statistical analysis in two steps. First, the statistical unit was the patients, and their characteristics were reported descriptively, using metrics of centrality and dispersion for quantitative data such as the mean and standard deviation whenever data proved to follow a normal distribution (Shapiro–Wilks test) and absolute frequencies with associated percentages for qualitative variables. Second, we considered the number of hospitalizations as the statistical unit, and we analyzed two groups with two or more hospitalizations during the time frame. Psychometric scores and quantitative characteristics that show deviations from normality were reported as the median and [Q1 to Q3] (Q1 reports the value of the 25th percentile and Q3 reports the values of the 75th percentile) with comparison made with the Wilcoxon test (admission vs. discharged) for paired groups and Mann–Whitney test for two independent groups. The box and whisker plot and density probability plot were used to represent the raw data. Statistical analysis was made with JASP (v. 0.18.3.0, Amsterdam, The Netherlands), considering a significance level of 5%; thus, the *p*-values less than 0.05 were considered statistically significant.

## 3. Results

### 3.1. Patients’ Characteristics

Sixty-two patients with 157 hospitalizations were evaluated, with 2 (45/62) to up to 7 hospitalizations. Hospitalizations were the highest in 2018 (35%) and 2019 (23.6%), with a few hospitalizations occurring in 2021 and 2022 (each year 12.1%). Patients had an age at first admission from 23 to 57 years, with half of them with an age from 31 to 40 years (31/62, 50%). The information regarding the age at diagnostic was available only for 28/62 patients, and varied from 17 to 56 years, with a mean of 27 years and a median of 24 years. Thirteen patients (21%) acknowledged unemployment while 24 (38.7%) were in ill-health retirement.

Most of the evaluated patients were male, living with someone, and not married (Table 2).

Most of the patients in our cohort did not acknowledge the presence of schizophrenia (35/62, 56.5%) or bipolar disorder (44/62, 71%) in their family history.

**Figure 2:**
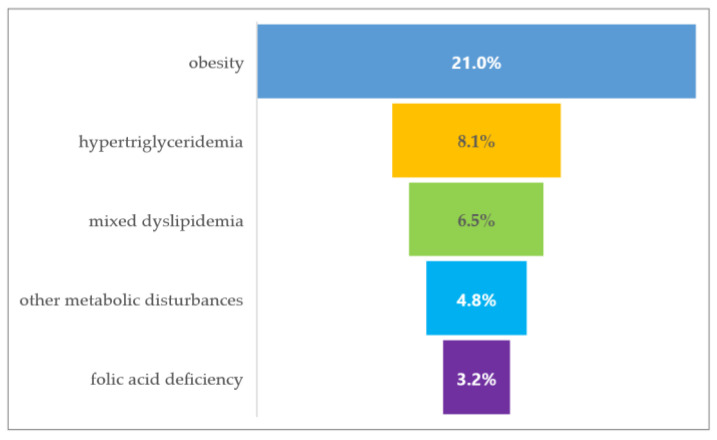
The frequency of metabolic comorbidities of the evaluated cohort.

Regarding associated comorbidities at first admission, four patients were with arterial hypertension, one reported ischemic cardiopathy, one reported hypertensive cardiopathy, and another one had carotid atheromatosis. Obesity was the most frequent comorbidity (Figure 2).

Almost 60% of evaluated patients declared one addiction (37, 59.7%) and five participants (8.1%) declared drugs consumption. The most frequent addiction was smoking (66.1%).

The discharged diagnostics were F20.0 (40/62, 64.5%), F20.3 (21/62, 33.9%), or F20.6 (1/62, 1.6%).

Most patients (72.6%) had two hospital admissions during the time frame of the study. The demographic data showed no statistically significant differences between patients with two hospital admissions and those with more hospital admissions (Age: *p*-value = 0.833 ^#^; Sex: *p*-value = 0.211 *; Living place: *p* = 0.092 *; Living condition: *p*-value = 0.929 *; Season of birth: *p* = 0.958 *; Married: *p*-value * = 0.187; * Fisher’s exact test, # Mann– Whitney test and Chi-squared test if no symbol).

**Figure 3:**
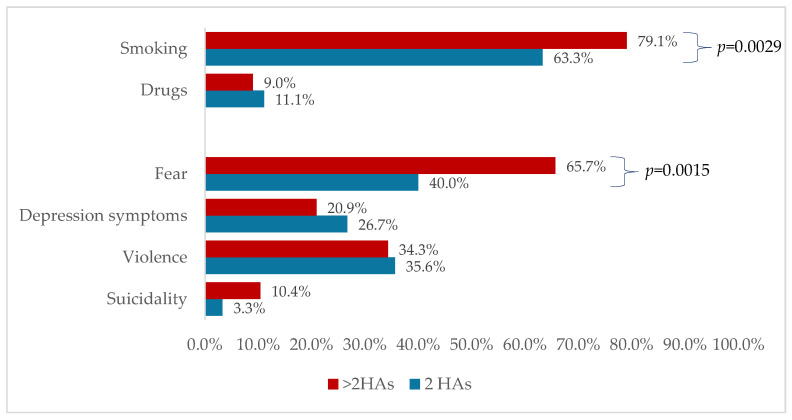
Reported symptoms and addictions at each admission by group (HAs = hospital admissions).

Fear as a symptom and smoking as an addiction were more frequently reported in the group with over two hospital admissions (Figure 3).

### 3.2. Psychometric Evaluation

**Figure 4:**
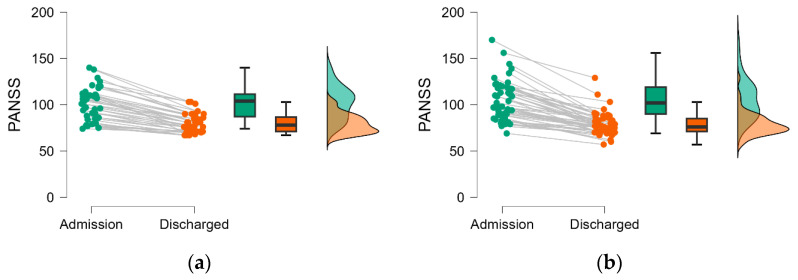
Changes in PANSS score at discharged compared to admission by hospitalization group: (**a**) patients with two hospital admissions; (**b**) patients with more than two hospital admissions. Dots are individual data, lines link the admission with the discharge, the box is constructed with the values of the 25th and 75th percentile, and the line in the box by the value of the median. The whiskers are given as the minimum and maximum.

**Figure 5:**
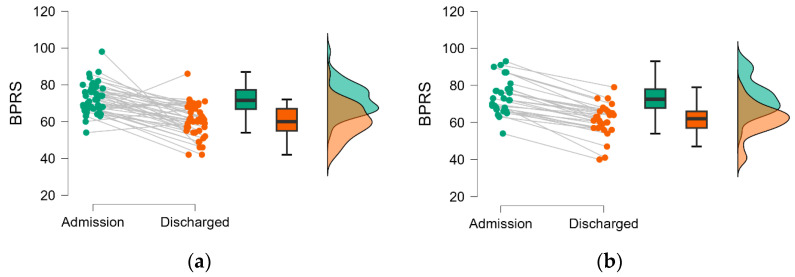
Changes in BPRS score at discharged compared to admission by hospitalization group: (**a**) patients with two hospital admissions; (**b**) patients with more than two hospital admissions. Dots are individual data, lines link the admission with the discharge, the box is constructed with the values of the 25th and 75th percentile and the line in the box by the value of the median. The whiskers are given as the minimum and maximum.

The PANSS scores (Figure 4) and BPRS (Figure 5) proved lower at discharge compared to admission regardless of the number of hospitalizations, and the differences reach the statistical significance threshold (Table 3).

### 3.3. Formatting of Mathematical Components

**Figure 6:**
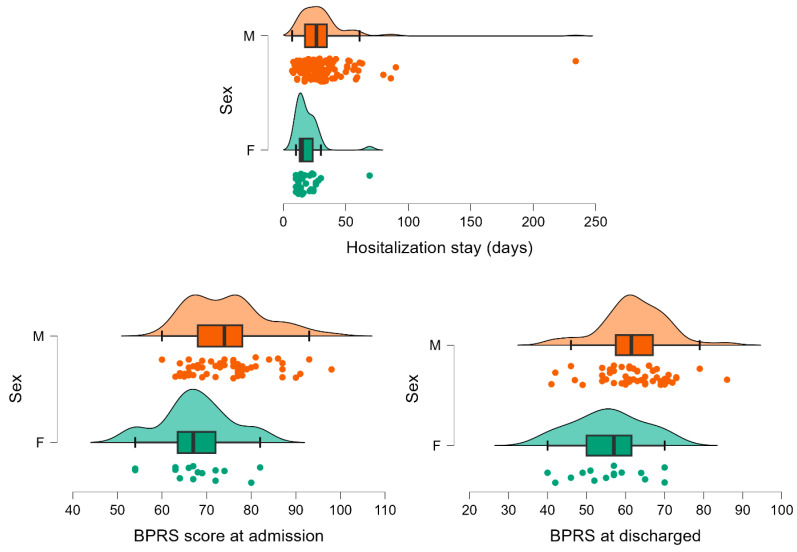
Distribution of hospitalization stay, and BPRS scores at admission and discharge by sex. Dots are individual data, lines link the admission with the discharge, the box is constructed with the values of the 25th and 75th percentile, and the line in the box by the value of the median. The whiskers are given as the minimum and maximum. M stands for male and F stands for female.

Statistically significant differences in hospitalization stays and BPRS at admission and discharge were observed between men and women (Table 4, Figure 6).

### 3.4. Therapy Schema and Reported Adverse Effects

At discharge, we had a lower percentage of three classes of medications, and a significantly smaller percentage of patients with FGA in the group with two hospital admissions compared to the group with over two hospital admissions (Table 5).

Half of the patients (54.8%) reported at least one adverse effect. Tachycardia occurred in 29% (33.3% vs. 17.9% in the group with two than over two hospital admissions, *p*-value = 0.0604), muscle stiffness in 27.4%, tremors in 16.1%, and ataxia in 9.7%, without significant differences between the groups based on the number of hospitalizations (*p*-values > 0.20). Ataxia occurred only in men (6/48, 12.5%). The occurrence of adverse effects was similar among women and men (*p*-values > 0.05).

## 4. Discussion

Our study found that most of the investigated patients are male with a mean age of 37 years, not married, and not living alone in urban settings. Patients with more hospitalization are more frequent smokers and had at admission fear symptoms. The investigated groups did not show significant differences in psychometric scores, but all patients experienced a significant improvement in the severity of symptoms upon discharge. Women stay less in hospital than men and have higher BPRS scores than men at admission and discharge.

The demographic characteristics of the investigated cohort regarding male predominance and average age of illness diagnosis (Table 2) are similar to other published reports on Romanian patients diagnosed with schizophrenia [18]. Most patients in our cohort experienced at least one addiction, with smoking being the most prevalent (Figure 3), results that are also consistent with those outlined in the specialty literature [22]. Although within our patient cohort, there are instances of family history related to schizophrenia or bipolar affective disorder, alluding to the potential for disease development through genetic transmission, most of the evaluated patients do not report a positive family history. Our result may suggest an independent nature of disease development in the presence of previously mentioned risk factors, findings that are consistent with those reported by Somnath et al. [23].

To the best of our knowledge, differences between patients with schizophrenia requiring two or more hospitalizations have not been investigated regarding the psychometric evolution from admission to discharge, using the overall PANSS or BPRS scale. We observed an improvement in symptomatology on the evaluated patients highlighted by statistically significant differences in the severity of both overall scale scores from admission to discharge, regardless of the frequency and recurrence of hospitalizations (see Table 3 and Table 4, Figure 4 and Figure 5). A statistically significant difference between men and women in BPRS scores at admission and discharge, but not in PANSS scores (Table 4 and Figure 6), was observed in our cohort. This result must be interpreted with caution and validated in an external cohort considering that the psychometric assessment is decided by the attending physician, and it is not imposed by healthcare practice. However, the difference observed only on a BPRS scale could be explained by the variation in comprehensiveness and item count between the two scales, findings consistent with those reported by Leucht et al. [24]. Nevertheless, the change in the overall severity scores on the psychotropic medication administered during hospitalization could explain discharged compared to admission, albeit not solely, medication found administered in different regimens at discharge in the two evaluated groups (Table 5). Specifically, the group with only two hospitalizations showed a notably low utilization rate of all three medication classes (FGAs, SGAs, and mood stabilizers/anticonvulsants as augmentation treatment), with an even lower prevalence of FGA at discharge (Table 5). It is known that antipsychotic medication can potentially result in tardive dyskinesia, which has been associated with increased rates of hospitalization among patients with schizophrenia [25]. Our findings might suggest the need to approach treatment strategies differently based on the recurrence of hospitalizations, which, in turn, could indicate the chronic and debilitating nature of the illness with a need to increase the level of assertiveness in the chosen treatment to achieve improvement similar to those with fewer hospitalizations.

Our study, while offering insights regarding the characteristics of patients with schizophrenia and multiple hospitalizations, is not without limitations. The first limitation could be attributed to the limited number of investigated patients, so the results reflect the sample and could not be generalized. The limited number of investigated patients also could explain the statistically significant differences observed between women and men in regard to the BPRS score. It should be noted that the decision for psychometric assessment belongs to the attending physician, and it is not a current practice. The investigation of a larger cohort, by extending the time frame or number of centers, following standardized psychometric assessment would more appropriately capture this particular population. The second limitation is related to the study design and retrospective collection of data. A more accurate picture would be obtained by using the same psychometric scale for all patients and all hospitalizations to decrease the heterogeneity of the psychometric assessment. The third limitation, in the same frame of the applied design, is represented by the access to only the overall score for psychometric PANSS assessment, so a prospective inclusion of patients in the study could withdraw this limitation. The fourth limitation is represented by the absence of evaluation of the pharmacokinetic interactions between psychotropic medication and other medication that individual patients received for different comorbidities.

## 5. Conclusions

Our results showed that the candidate for multiple hospitalizations is a male, with a mean age of 37 years, unmarried, and living with someone in urban settings, more likely a smoker who exhibits fear symptoms. No significant differences in psychometric scores between those with two and those with more hospitalizations, but all patients showed a significant improvement in symptom severity by the time of discharge. Additionally, women had shorter hospital stays compared to men and exhibited different Brief Psychiatric Rating Scale scores, both at admission and discharge, but these findings need to be confirmed on an external cohort. If our findings will be externally confirmed, we would suggest a need for tailored interventions considering sex differences and lifestyle factors, such as smoking in the management of hospitalized patients with schizophrenia.

## Figures and Tables

**Table 1:** Questionnaires’ description and interpretation.

Abbreviation—Full Name [Ref.]	Description	Interpretation
SCI-PANSS/PANSS—Structured clinical interview for the positive and negative syndrome scale [20]	30 items 7 items for positive symptoms, score from 7 to 49 7 items for negative symptoms, score from 7 to 49 16 items for symptoms of general psychopathology, score from 16 to 122	bipolar index = (positive score)/(negative score) ranges from −42 to +42, which is essentially a reflective difference score of the degree of predominance of one syndrome in relation to the other
BPRS—brief psychiatry rating scale [21]	18 items correspond to the semiological consensus components: somatic concerns, anxiety, emotional withdrawal, disorganization conceptual, feelings of guilt, tension, mannerisms and postures, grandiosity, depressed mood, hostility, suspiciousness, hallucinatory behavior, motor slowness, uncooperativeness, unusual ideational content, affective dulling, excitement, and disorientation	0–9 → absence of any syndrome 10–20 → minor syndrome ≥21 → major syndrome

**Table 2:** Cohort characteristics.

Variable	Level of Measurement	All Samples of Patients, n = 62
Age at admission ^(a)^	Years	37 (8.1)
Sex ^(b)^	Female Male	14 (22.6) 48 (77.4)
Living place ^(b)^	Rural Urban	29 (46.8) 33 (53.2)
Lives ^(b)^	Alone With somebody	13 (21) 44 (71)
Season of birth ^(b)^	Winter Spring Summer Autumn	14 (22.6) 14 (22.6) 15 (24.2) 19 (30.6)
Married ^(b)^	Yes No	8 (12.9) 40 (64.5)

^(a)^ data described by mean (standard deviation); ^(b)^ data described by absolute frequency (relative frequency, %).

**Table 3:** Psychometric scores by hospitalization.

KERRYPNX	All Patients	2 Hospitalizations	>2 Hospitalizations	*p*-Value ^*^
Age at admission, years	36.5 [33 to 44]	37 [33 to 44]	33 [33 to 44.5]	0.833
Hospitalization stays, days	24 [16 to 33]	24 [16 to 34.8]	16 [16 to 32]	0.993
PANSS				
Admission	103 [89 to 117.3]	102 [90 to 119]	87 [87 to 111.5]	0.467
Discharged	77 [71 to 86]	76 [71 to 85]	71 [71 to 86.5]	0.665
*p*-value ^#^	<0.001	<0.001	<0.001	
PANNS % score reduction	18.5 [14 to 26]	19 [14 to 27]	13.5 [13.5 to 25]	0.338
BPRS				
Admission	72 [67 to 78]	71.5 [66.8 to 77.3]	67.75 [67.8 to 78]	0.506
Discharged	61 [56 to 67]	60 [55 to 67]	57 [57 to 66]	0.549
*p*-value ^#^	<0.001	<0.001	<0.001	
BPRS % score reduction	16 [12 to 20]	17 [12 to 20]	12 [12 to 20]	0.941

Data are reported as median [Q1 to Q3], where Q is the quartile; * Mann–Whitney test; ^#^ Wilcoxon test.

**Table 4:** Age, hospitalization stay, and psychometric scores by sex.

	Women	Men	*p*-Value *
Hospitalization stays, days	15 [13 to 23.5]	17.25 [17.3 to 34.8]	<0.001
Age, years	37 [34 to 44]	29.5 [29.5 to 44.5]	0.575
PANSS			
Admission	103 [90 to 119.3]	87.5 [87.5 to 112.5]	0.619
Discharged	76.5 [71 to 83.5]	70.75 [70.8 to 87]	0.082
No.	16	72	
*p*-value #	<0.001	<0.001	
PANNS % score reduction	19 [15 to 27]	13 [13 to 25]	0.178
BPRS			
Admission	70.5 [66 to 77.3]	74.5 [68 to 78]	0.012
Discharged	60 [54.3 to 66.5]	63 [59.5 to 66.5]	0.016
No.	15	54	
*p*-value #	<0.001	<0.001	
BPRS % score reduction	17 [12 to 20]	12.5 [12.5 to 19.5]	0.315

Data are reported as median [Q1 to Q3], where Q is the quartile; * Mann–Whitney test; ^#^ Wilcoxon test; No.—number of psychometric evaluations.

**Table 5:** Therapy schemas and adverse effects.

		All Patients	2 Hospitalizations	>2 Hospitalizations	*p*-Value
No. classes of medications	Admission				0.252
	1	17 (10.8)	11 (12.2)	6 (9)	
	2	101 (64.3)	61 (67.8)	40 (59.7)	
	3	39 (24.8)	18 (20)	21 (31.3)	
	Discharged				<0.001
	1	33 (21)	20 (22.2)	13 (19.4)	
	2	97 (61.8)	57 (63.3)	40 (59.7)	
	3	26 (16.6)	12 (13.3)	14 (20.9)	
FGA	Admission	57 (36.3)	29 (32.2)	28 (41.8)	0.218
	Discharged	41 (26.3)	17 (19.1)	24 (35.8)	0.019
SGA	Admission	147 (93.6)	85 (94.4)	62 (92.5)	0.628
	Discharged	154 (98.7)	88 (98.9)	66 (98.5)	0.753 *
Adjuvant	Admission	132 (84.1)	73 (81.1)	59 (88.1)	0.239
	Discharged	110 (70.5)	65 (73)	45 (67.2)	0.426

Data are reported as number (%); *p*-value resulted from the application of Chi-squared test, except * when the Fisher’s exact test was applied; FGA = first generation antipsychotics; SGA = second generation antipsychotics.

## Data Availability

The original contributions presented in the study are included in the article, and further inquiries can be directed to the first author.
